# An exploratory pilot study evaluating the supplementation of standard antibiotic therapy with probiotic lactobacilli in south African women with bacterial vaginosis

**DOI:** 10.1186/s12879-019-4425-1

**Published:** 2019-09-18

**Authors:** Harold Marcotte, Per Göran Larsson, Kasper Krogh Andersen, Fanglei Zuo, Lasse Sommer Mikkelsen, Erik Brandsborg, Glenda Gray, Fatima Laher, Kennedy Otwombe

**Affiliations:** 1 Department of Laboratory Medicine, Division of Clinical Immunology and Transfusion Medicine, Karolinska Institutet at Karolinska University Hospital Huddinge, 14186 Stockholm, Sweden; 2 Department of Obstetrics and Gynaecology Kärnsjukhuset, Skaraborg Hospital, 54185 Skövde, Sweden; 3 Department of Clinical and Experimental Medicine (IKE), Linköping University, 58183 Linköping, Sweden; 4 Bifodan A/S, 3390 Hundested, Denmark; 5 Perinatal HIV Research Unit, Faculty of Health Sciences, University of the Witwatersrand, Johannesburg, 1862 South Africa

**Keywords:** *Lactobacillus*, Bacterial vaginosis, Probiotics, *Lactobacillus rhamnosus* DSM 14870, *Lactobacillus gasseri* DSM 14869

## Abstract

**Background:**

To reduce acquisition and relapse of bacterial vaginosis (BV), lactobacilli must be maintained in the vaginal microbiome. Probiotic lactobacilli may aid this purpose. We investigated whether vaginal probiotics (containing *Lactobacillus rhamnosus* DSM 14870 and *Lactobacillus gasseri* DSM 14869) would result in vaginal colonisation with lactobacilli in women with and without BV.

**Methods:**

This prospective, partially randomised, exploratory pilot study was conducted in Soweto, South Africa. Thirty-nine sexually-active, HIV negative women were enrolled from October 2014 to May 2016 into three arms. Women who did not have BV (Group 1, *n* = 13) self-administered probiotic capsules vaginally once daily for 30 days, then once a week until Day 190. Women diagnosed with BV were randomized into Group 2 (*n* = 12) or Group 3 (*n* = 14) and treated with the triple oral antibiotic combination for vaginal discharge syndrome per South African guidelines (cefixime 400 mg stat, doxycycline 100 mg BD for 7 days and metronidazole 2 g stat). Immediately after antibiotic treatment, women in Group 2 self-administered probiotic capsules vaginally once daily for 30 days then vaginally once a week until Day 190. Women in Group 3 were not given lactobacilli.

**Results:**

During the study, *L. rhamnosus* DSM 14870 or *L. gasseri* DSM 14869, were isolated in 5/13 (38.5%) women in Group 1 compared to 10/12 (83.3%) women in Group 2 (*p* = 0.041). The 1-month and 6-month BV cure rates were similar (*P* >  0.05) between Group 2 (42 and 25%) compared to Group 3 (36 and 25%). In Group 2, no correlation was observed between the frequency of isolation of the two *Lactobacillus* strains and the 1-month or 6-month cure rate.

**Conclusions:**

Supplementation with vaginal probiotic capsules resulted in colonisation of the vagina by the *Lactobacillus* strains (*L. rhamnosus* DSM 14870 and *L. gasseri* DSM 14869) contained in the capsules. We observed low initial cure rates of BV after a stat dose of metronidazole and that the probiotic did not improve BV cure rates or alleviate recurrence which could be due to treatment failure or very limited power of the study.

**Trial registration:**

Registered at the Pan African Clinical Trial Registry (www.pactr.org) on April 13, 2018 (retrospectively registered). Trial identification number: PACTR201804003327269.

**Electronic supplementary material:**

The online version of this article (10.1186/s12879-019-4425-1) contains supplementary material, which is available to authorized users.

## Background

Bacterial vaginosis (BV) is characterised by a change in the vaginal ecosystem where levels of *Lactobacillus* species are strikingly reduced, while the proportion of anaerobic microorganisms like *Gardnerella vaginalis*, *Mycoplasma hominis*, *Prevotella* species, *Mobiluncus* species and Clostridia species are greatly increased [1, 2]. It is regarded as a mild illness but with potentially serious consequences such as preterm births, pelvic inflammatory disease and higher risk of acquiring a sexually transmitted infection such as Human Immunodeficiency Virus (HIV) [3–5]. BV manifests clinically as a thin, whitish, homogeneous vaginal discharge and the presence of an amine odour [6].

BV is diagnosed based on Amsel clinical criteria or microscopic observation of Gram-stained vaginal smear (Nugent scoring method) [7]. The current treatment regimens include either vaginal clindamycin, oral/vaginal metronidazole or tinidazole [8]*.* The recurrence rate following antibiotic treatment is high, as shown by a large number of treated cases experiencing relapses. Trials conducted in North America, Europe and Australia reported initial 1-month cure rate of 80–90% following antibiotic treatment, but recurrence rates of 40% within 3 months [9, 10].

Estimates of BV prevalence in the African population range from 20 to 50% with higher levels documented in female sex workers [11–13]. The high prevalence of BV could be an important factor contributing to the high prevalence of HIV in sub-Saharan Africa [3, 4]. The World Health Organisation recommends a syndromic diagnostic approach for the management of sexually transmitted infection (STI) and symptomatic BV in low-income countries where routine diagnostic tests are unavailable [14]. Syndromic management refers to the approach of treating STI symptoms and signs based on the organisms most commonly responsible for each syndrome. In South Africa, patients presenting with vaginal discharge syndrome are treated with a combination of three antibiotics including cefixime (400 mg po stat) or ceftriaxone (250 mg imi stat) for treatment of *Neisseria gonorrhea*, doxycycline (100 mg BD po for 7 days) for treatment of *Chlamydia trachomatis,* and metronidazole (2 g po stat) for treatment of *Trichomonas vaginalis* and BV [15]. However, 90% of BV cases in South Africa are asymptomatic and vaginal discharge is a poor indicator of BV [16]. Furthermore, high recurrence rates (> 70% after 3 months) of clinically defined BV after metronidazole therapy was confirmed in a few studies in Africa [12].

In order to reduce the prevalence and relapse of BV and associated risk of sexually transmitted diseases, a normal vaginal microbiome must be maintained. *Lactobacillus*-dominance of the human vaginal microbiome is hypothesised to benefit women by reducing disease risk. Lactobacilli protect the host from urogenital infections by maintaining a low pH (< 4.5), by producing bacteriostatic and bactericidal substances and through competitive exclusion. More than 70 % of healthy Caucasian females carry predominantly vaginal lactobacilli with the most commonly identified *Lactobacillus* species being *L. crispatus, L. gasseri, L. jensenii*, *L. iners* and *L. vaginalis* [2, 17, 18]. The composition of the vaginal microbiome may also be influenced by genetic factors as evidenced by differences in bacterial communities observed in asymptomatic North American women representing 4 racial/ethnic groups. *Lactobacillus*-dominated communities were found in 90% of Caucasian, 80% of Asian, 65% of Hispanic, and 61% of Black women [2]. The few studies focused on sub-Saharan African countries indicated that the predominant *Lactobacillus* species varied both within and between countries [19]. Overall, 40 to 75% of healthy, asymptomatic African women carry predominantly *Lactobacillus* with *L. iners* and *L. crispatus* being the most dominant followed by *L. vaginalis, L. gasseri* and *L. jensenii* [20–25].

Several clinical trials have been performed to investigate whether specific strains of lactobacilli, administered vaginally or orally, in combination with antibiotics or not, are able to colonise the vagina of women with BV and improve symptoms [26, 27]. *L. gasseri* DSM 14869 and *L. rhamnosus* DSM 14870 have been shown to colonise the vagina of Scandinavian women and reduce relapses in BV patients by about 20% over six months when administered vaginally [26–28]. However, very few clinical studies have evaluated the efficacy of supplement with lactobacilli to help maintain a healthy vagina in African women [29, 30] and to our knowledge, none have previously tested vaginal probiotic capsules. Supplementation of standard antibiotic therapy with vaginal probiotics could improve the cure rate of BV and reduce the relapse rate in sub-Saharan African countries. Furthermore, a successfully colonising *Lactobacillus* could be further engineered for delivery of a microbicide (defined as a substance that destroys a microbe of interest, commonly HIV) in the vagina combining both the specificity of the microbicide with the probiotic activity of the lactobacilli [31]. In this study conducted in South Africa, we tested if vaginal administration of probiotic capsules in women with normal microbiota and women with BV results in colonization with *L. gasseri* DSM 14869 and *L. rhamnosus* DSM 14870 and improvement of the efficacy of the standard antibiotic treatment of BV.

## Method

### Study setting, population and study group

This prospective, partially randomized, open label, exploratory, pilot study was conducted at the Perinatal HIV Research Unit (PHRU), Chris Hani Baragwanath Hospital, in Soweto, South Africa in women that were enrolled between October 2014 and May 2016. The study enrolled sexually active women into one of three arms: women who did not have BV (“healthy”) were automatically assigned to receive probiotic capsules (Group 1), and women presenting with BV were enrolled to either Group 2 to receive both antibiotics and probiotic capsules or to Group 3 for antibiotics alone.

To be eligible, participants had to be female, 18–40 years, pre-menopausal on menstrual history, able to read and understand English, provide voluntary informed consent, HIV-negative, have an intact genital tract, use Depot Medroxyprogesterone Acetate or norethisterone enanthate injectable contraception for at least two months prior to study entry, and be willing to receive HIV testing and counselling. Participants were excluded from the study if they were positive for pregnancy test or breastfeeding at study entry, were infected with *C. trachomatis*, *Neisseria gonorrhea* or *T. vaginalis*, showed history of abnormality on Pap test, were currently using oral contraceptive, were menstruating on enrolment, did not have sexual debut, were treated with antibiotics within eight weeks of study enrolment, were using oral or vaginal probiotic capsules within eight weeks of enrolment in the study.

### Data and sample collection at study entry

Socio-demographic data and gynaecologic status were filed on day − 7 (visit 0). Social-demographic data included age, education, occupational status, marital status, housing, and tobacco and alcohol use. The gynaecologic status included first day of last normal menstrual period, method of contraception, current symptoms of BV (vaginal discharge and presence of amine odour), previous treatment for BV and sexually transmitted diseases (STI), sexual history (partners, age of sexual debut, use of condom) and vaginal hygiene practice (douching, use of soap).

Gynaecologic examination was conducted using a speculum. One swab specimen was collected to extract DNA for determining *C. trachomatis* and *N. gonorrhoea* infection using PCR according to the local laboratory routine. One vaginal cotton swab sample (Copan Venturi Transsystem®, Copan Diagnostics, Italy) was used to prepare a dried smear on a glass slide for scoring of BV by the Nugent and modified Hay/Ison methods. The swab was subsequently put in Amies agar gel medium with charcoal for cultivation of lactobacilli (see below). Blood specimens were collected for HIV testing.

### Lactobacilli used for treatment

Vaginal capsules containing probiotic strains *L. gasseri* DSM 14869 and *L. rhamnosus* DSM 14870 at 1 × 10^8^ CFU of each strain/capsule were produced by Bifodan A/S, Denmark.

### Enrolment and treatment

**Fig. 1:**
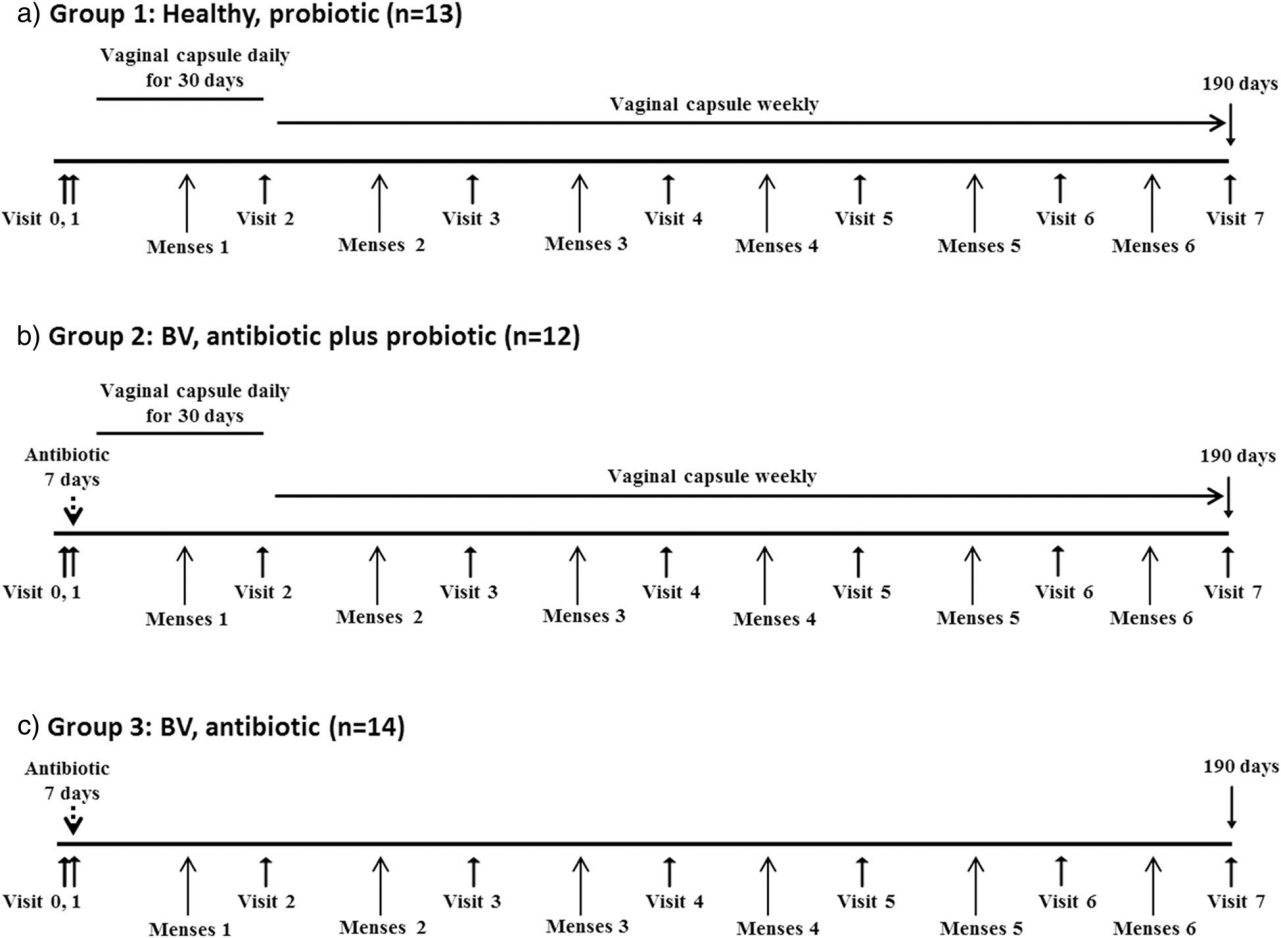
Time schedule of the treatment of BV and follow-up. **a**) Group 1, women who did not have BV (“healthy”) receiving probiotic capsules, **b**) Group 2, women presenting with BV receiving both antibiotics and probiotic capsules, **c**) Group 3, women presenting with BV receiving antibiotics only. Sociodemographic data and gynaecologic status were filed on visit 0. On visit 1, volunteers were enrolled and given the prescription. Enrolment into each arm was stratified by age-group: 18–29 and 30–40 years, using a blocked randomisation strategy. Microscopic scoring of BV and cultivation of lactobacilli were performed on visit 0, and 2 to 7

On visit 1 (day 0), following completion of exclusion and inclusion criteria as well as gynaecological history, patients were enrolled by the study nurse and given the prescription. Enrolment into each arm was stratified by age-group: 18–29 and 30–40 years, using a blocked randomisation strategy that was computer generated by the study statistician. Healthy women were required to self-administer probiotic capsules vaginally once daily for 30 days thereafter once a week until Day 190. All women with BV took a combination of three antibiotics orally for vaginal discharge syndrome as per South African guidelines i.e. cefixime (400 mg stat), doxycycline (100 mg twice daily for 7 days) and metronidazole (2 g stat). Immediately after the antibiotic treatment, women in group 2 self-administered probiotic capsules vaginally once daily for 30 days (i.e. day 0 to 30) followed by capsules vaginally once a week until Day 190 (Fig. 1).

### Follow-up

Participants attended one visit per month for six months (visits 2–7), with an interval of between 3 and 5 weeks, at the PHRU in Chris Hani Baragwanath Hospital until day 190 (visit 7). The primary outcome measure of the study was colonisation by *Lactobacillus* strains contained in the probiotic capsules (*L. rhamnosus* DSM 14870 and *L. gasseri* DSM 14869) while the secondary outcome measure was to evaluate the effect of supplementation of antibiotic therapy with probiotic strains on curing BV and preventing relapse. At each visit, data were collected on socio-demographic factors and gynaecological history. One cotton vaginal swab was used to prepare a vaginal smear and subsequently put in the Amies transport medium for bacterial cultivation. HIV blood test was performed on visit 7.

### Microscopic scoring of BV

The Nugent and modified Hay/Ison scoring of BV was performed in a blinded manner by the clinician. On visit 0 (screening), the smears were either scored in PHRU by an experienced Swedish clinician (PGL) who travelled to South Africa, or initially scored by a South African clinician and the slides immediately sent to Skaraborgs hospital Skövde and Linköping University, Sweden for confirmation of the results. In the latter case, the participants were assigned to each treatment group following scoring of the smears in PHRU, and withdrawn if the score was not confirmed by the Swedish clinician. On visit 2 to 7, the air-dried vaginal smears were sent to Sweden for scoring. First, the vaginal air-dried smears were rehydrated and scored according to modified Hay/Ison into either normal (1) intermediate (2) or BV (3) [32, 33]. Then, the smears were Gram stained and the number of different bacterial morphotypes in Gram stained smears were counted in high power fields using microscopy with a 1000× magnification. Each slide was classified according to Nugent score classification to either normal (≤ 3), intermediate (4–6) or BV (≥ 7) [7]. Women were included in the healthy group (Group 1) if the Nugent score was ≤3 and in the BV groups (Group 2 and 3) if ≥7. Women with intermediate scores (4–6) were excluded from the study in order to reduce variability. For Group 2 and 3, the 1-month cure rate was defined as the percentage of women having a Nugent score < 7 at visit 2 while the 6-month cure rate was calculated as the proportion of women with Nugent scores < 7 for the entire follow-up (visits 2–7) (also including participants who did not provide all the samples during follow-up). For each treatment group, relapse rate was defined as the proportion of women having a relapse (Nugent score ≥ 7) at any visit following initial cure at visit 2.

### Sample processing

#### Cultivation of lactobacilli

The swabs in Amies agar gel medium were transported via courier at room temperature to the laboratory in Karolinska Institutet, Sweden within a maximum of three days. Upon arrival, vaginal swabs (visits 0, and 2 to 7) were directly streaked onto three of each Rogosa agar (BD Difco™ Rogosa SL agar, Becton, Dickinson and Company, Spark, MD) and Columbia agar (BD Difco™ Columbia Blood Agar Base) with 5% horse blood. Plates were incubated for 48 h at 37 °C in anaerobic condition using BD GasPack™ EZ gaz generating systems (Becton, Dickinson and Company). Colonies with *Lactobacillus* morphology and yielding bacilli were re-streaked. Respective isolated colonies from re-streaked plates were used to inoculate MRS broth medium. Tiny transparent colonies (*L. iners*-like) from blood agar plates yielding Gram-positive bacilli were directly collected from the plates and were used for genomic DNA isolation. Glycerol stocks (15%) were prepared and stored at − 80 °C. Five to ten colonies (generally 8) were picked per samples.

#### Identification of isolates to the genus Lactobacillus

Genomic DNA was extracted from all cultured isolates using Invisorb Universal Bacteria HTS 96/V (STRATEC Molecular GmbH, Berlin, Germany). Molecular identification of *Lactobacillus* genus was carried out by PCR using the previously designed *Lactobacillus* 16S ribosomal RNA (rRNA) specific primers L (also previously referred to as for-lac and Lactobac F in the literature) (5′-TGGAAACAGGTGCTAATACCG-3′) and R (also previously referred to as Rev-lac and Lactobac R) (5′-CCATTGTGGAAGATTCCC-3′) [34–37] (Additional file 1: Table S1). The primers were blasted against the 16S rRNA sequence database and both primer sequences were found to be homologous to the 16S rRNA gene of *Lactobacillus* vaginal strains of different species (*L. crispatus*, *L. gasseri*, *L. vaginalis*, *L. jensenii* and *L. iners*). Only one mismatch was observed between primer F (position 9) and the 16S rRNA gene of some vaginal strains of *L. gasseri* (including *L. gasseri* DSM 14869), *L. iners* and *L. rhamnosus* (including *L. rhamnosus* DSM 14870). The genomic DNA from *L. rhamnosus* DSM 14870 and *L. gasseri* DSM 14869 was always included as a positive control (in duplicate) for all the PCR assays. The primers were tested against 64 *Lactobacillus* strains of various species including vaginal strains previously isolated from South Africa (Additional file 1: Table S2) and all tested positive (Additional file 1: Figure S2).

The amplification mixture contained 25 ng template DNA, 1 μM of each primer, 1 X Green GoTaq™ Flexi reaction buffer (Promega, Madison, Wisconsin, USA), 200 μM of each dNTP, 3 mM MgCl_2_, and 1.5 U GoTaq™ DNA Polymerase in a total volume of 25 μl. The PCR was performed under the following conditions: 94 °C for 3 min; 30 cycles of 94 °C for 30 s, 57 °C for 30 s and 72 °C for 30 s, and a final extension step at 72 °C for 7 min.

#### Identification of *L. rhamnosus* DSM 14870 and L. gasseri DSM 14869

For identification of *L. rhamnosus* DSM 14870 and *L. gasseri* DSM 14869 to the strain level, a multiplex PCR consisting of amplification of the 16S rRNA gene (1.5 kb) using the eubacterial primers P0 (5′-GAGAGTTTGATCCTGGCTCAG-3′) and P6 (5′-CTACGGCTACCTTGTTACGA-3′) (positive control) [20, 38, 39] combined with strain specific primers designed on CRISPR spacer array was developed. The P0 and P6 primers were previously used for identification of *Lactobacillus* strains isolated from the vagina of South African women using amplification and sequencing of 16S rRNA gene [20]. The CRISPR array sequences were obtained from the published *L. rhamnosus* DSM 14870 and *L. gasseri* DSM 14869 genome sequence (Additional file 1: Figure S1) [40]. Blast alignment of the whole CRISPR regions as well as individual primers was performed to ensure the specificity of the primer pairs. The primer pairs selected for *L. rhamnosus* DSM 14870 consist of scr-Rhmn-2Fw (5′-AAAGTCGTGTATATGTAGCCGG-3′) and scr-Rhmn-2Rv (5′-GAACCGTCTCGTCTTCCAATAG-3′) while the primers specific for *L. gasseri* DSM 14869 consist of primers scr-gass-1Fw (5′-CCTATTGTCGAACCCTTACGAA-3′) and scr-gass-1Rv (5′-GTGCTGAAATGCTTGGTGTAG-3′) (Additional file 1: Table S1). Each of the primers, scr-Rhmn-2Fw and scr-gass-1Rv, had DNA sequence homology only with another *Lactobacillus* genome (*L. rhamnosus* LOCK900 and *L. acidophilus* NCTC13720 respectively) while the scr-Rhmn-2Rv and scr-gass-1Fw primers show only partial sequence homology with the genome of other *Lactobacillus* species or other bacterial genera.

Amplification reactions were performed in a total volume of 25 μl containing 25 ng of template DNA and a reaction mix of 20 mM Tris–HCl, 50 mM KCl, 200 μM of each deoxynucleoside triphosphate, 0.5 μM of each of the four primers (P0/P6 combined with the pair scr-Rhmn-2Fw/scr-Rhmn-2Rv or scr-gass-1Fw/scr-gass-1Rv), 1.5 mM MgCl_2_ and 1.5 U of *Taq* DNA polymerase (Gibco-BRL Life Technologies, UK).

The PCR was performed under the following conditions: 94 °C for 3 min; 30 cycles of 94 °C for 30 s, 57 °C for 30 s and 72 °C for 2 min, and a final extension step at 72 °C for 7 min.

Genomic DNA from *L. rhamnosus* DSM 14870 and *L. gasseri* DSM 14869 was used as positive control in each PCR assay. The PCR was also tested against 64 *Lactobacillus* strains of different species (Additional file 1: Table S2) and all tested negative, showing the specificity of the method for detection of *L. rhamnosus* DSM 14870 and *L. gasseri* DSM 14869 (Additional file 1: Figures S3 and S4).

#### Ethical considerations

Written consent was obtained from each participant for their information to be stored in the clinic database and used for research purposes. The ethical approval for this study was obtained from the University of the Witwatersrand Human Research Ethics Committee (Medical).

Participation in the study was voluntary and participants could withdraw or decline participation at any time without having to state reasons. Participants who withdraw were still able to access the standard of medical care.

There were no direct benefits of being in the study but standard BV antibiotics, HIV counseling and testing were offered on this study, though it is also available at local clinics. Participants were referred for local care if they decline or withdraw from participation. The participant’s information was kept confidential. Each participant was assigned a unique study identification number. This number was recorded on each case report form (CRF) and clinical specimen to facilitate linkage of data.

The clinical trial was registered on April 13, 2018 at the Pan African Clinical Trial Registry (www.pactr.org) with the trial identification number PACTR201804003327269.

#### Sample size

This was an exploratory pilot study designed to determine whether *L. gasseri* DSM 14869 and *L. rhamnosus* DSM 14870 contained in the probiotic capsules can colonise the vagina of healthy and BV infected women. The aim was to recruit a total of 39 participants divided equally between the three groups (*n* = 13 per group).

#### Statistical analysis

Data were analysed in SAS Enterprise Guide 7.1 using the SAS/STAT. Frequencies were determined for categorical data while descriptive measures such as medians, interquartile ranges (IQR) and means were determined for continuous measures. Since BV incidence was assessed at each visit during follow-up, factors associated with BV were determined longitudinally using the inverse probability weighted generalised estimating equations (GEE). The weights were used to account for missing data (i.e to account for those missing their visits because of withdrawal of consent or loss to follow-up). Weights were determined from cumulative probabilities at each visit (that were based on the number of participants attending) followed by calculation of their inverse. Since BV seemed to occur greater than 10% in the BV arms during follow-up, risk of BV was assessed by relative risk (RR) and its associated 95% confidence interval. At enrolment, all the participants were asked whether they had ever been treated for BV in the past 12 months. Univariate analysis was conducted in which variables with a *p*-value ≤0.1 were considered for inclusion in the multivariate model. Thereafter, variables were reduced using the stepwise selection procedure [41]. Variables that were significant at the univariate level or plausible were included in the multivariate model.

The frequency of isolation was determined as the percentage of samples positive for *L. rhamnosus* DSM 14870 and/or *L. gasseri* DSM 14869 (or other lactobacilli) on the total number of samples in each group. The frequency of isolation was calculated at each visit and for the entire study (visits 2 to 7). The difference in frequency of isolation of *L. rhamnosus* DSM 14870 and *L. gasseri* DSM 14869 or other lactobacilli between groups was compared by Fisher’s exact test using a p-value cutoff of 0.05 (two tailed).

## Results

### Socio-demographic data and gynaecological history

**Fig. 2:**
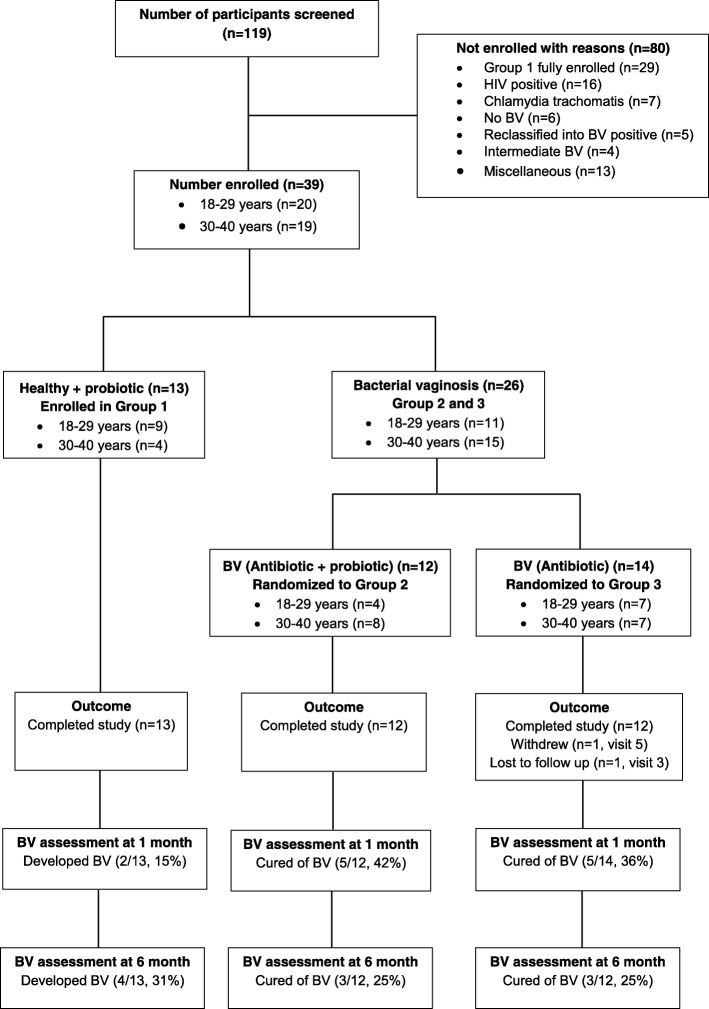
Participant disposition flow chart

Of the 119 screened women, 39 were enrolled into three arms: healthy women who were assigned to receive vaginal probiotic capsules (Group 1, *n* = 13), women presenting with BV who were assigned to receive both antibiotics and vaginal capsules (Group 2, *n* = 12) and women presenting with BV who were assigned to receive antibiotics (Group 2, *n* = 14) (Figs. 1 and 2). Thirty-seven out of 39 (95%) enrolled women completed the study (Fig. 2). Women in Groups 1, 2 and 3 spent a median of 182 (IQR: 180–195), 182 (IQR: 181–182) and 180 (IQR: 175–180) person days of follow-up.

Whereas unbalanced numbers were observed in some of the variables at enrolment, there were no significant differences in most of them (Tables 1 and 2). In Group 1, most women were 18–29 years old (69%) whereas in Groups 2 and 3, most were 30–40 years old (58%) (Table 1). Most participants had attained secondary level education (85%), were unemployed (77%) and had never been married (80%) (Table 1). There were 31 and 62% of women that reported use of tobacco and alcohol in the past 6 months respectively. Healthy participants (Group 1) were significantly more likely to be unemployed relative to BV participants receiving antibiotic and probiotic (Group 2) (84.6% vs 41.7%, *p* = 0.0254, Table 1).

**Table 1:** Socio demographic characteristics per study groups

Variables	Overall	Group 1 Healthy + probiotic	Group 2 BV + antibiotic + probiotic	Group 3 BV antibiotic
No. of enrolment	39	13	12	14
Age				
18–29 (%)	20 (51.28)	9 (69.23)	4 (33.33)	7 (50.00)
30–40 (%)	19 (48.72)	4 (30.77)	8 (66.67)	7 (50.00)
Median (IQR)	26.0 (22.0–31)	24.0 (23.0–31)	30.0 (24.5–32)	23.5 (20.0–30)
Highest level of education				
Secondary (%)	33 (84.62)	10 (76.92)	10 (83.33)	13 (92.86)
Tertiary (%)	6 (15.38)	3 (23.08)	2 (16.67)	1 (7.14)
Occupational status^a^				
Employed (%)	9 (23.08)	1 (7.69)	5 (41.67)	3 (21.43)
Student (%)	4 (10.26)	1 (7.69)	2 (16.67)	1 (7.14)
Unemployed (%)	26 (66.67)	11 (84.62)	5 (41.67)	10 (71.43)
Marital status				
Divorced (%)	3 (7.69)	0 (0.00)	3 (25.00)	0 (0.00)
Married (%)	5 (12.82)	2 (15.38)	0 (0.00)	3 (21.43)
Never married (%)	31 (79.49)	11 (84.62)	9 (75.00)	11 (78.57)
People living in a household				
1–5 (%)	26 (66.67)	10 (76.92)	7 (58.33)	9 (64.29)
> 5 (%)	13 (33.33)	3 (23.08)	5 (41.67)	5 (35.71)
Median (IQR)	5.00 (4.00–7)	5.00 (4.00–5)	5.00 (3.50–6.5)	5.00 (4.00–7)
Rooms in household				
1–3 (%)	14 (35.90)	5 (38.46)	6 (50.00)	3 (21.43)
> 3 (%)	25 (64.10)	8 (61.54)	6 (50.00)	11 (78.57)
Median (IQR)	4.00 (3.00–6)	4.00 (3.00–5)	3.50 (3.00–7)	4.00 (4.00–6)
House material				
Brick house (%)	34 (87.18)	11 (84.62)	10 (83.33)	13 (92.86)
Shack (%)	5 (12.82)	2 (15.38)	2 (16.67)	1 (7.14)
Used tobacco in the last 6 months				
No (%)	27 (69.23)	10 (76.92)	8 (66.67)	9 (64.29)
Yes (%)	12 (30.77)	3 (23.08)	4 (33.33)	5 (35.71)
Used alcohol in the last 6 months				
No (%)	15 (38.46)	7 (53.85)	4 (33.33)	4 (28.57)
Yes (%)	24 (61.54)	6 (46.15)	8 (66.67)	10 (71.43)

^a^ Healthy participants (Group 1) were significantly more likely (p = 0.0254) to be unemployed relative to BV participants receiving antibiotic (Group 2)

**Table 2:** Gynaecologic status per study groups at enrolment

Variables	Overall	Group 1 Healthy + probiotic	Group 2 BV + antibiotic + probiotic	Group 3 BV antibiotic
No. of enrolment	39	13	12	14
Currently have vaginal abnormal discharge^a^				
No (%)	25 (64.10)	12 (92.31)	6 (50.00)	7 (50.00)
Yes (%)	14 (35.90)	1 (7.69)	6 (50.00)	7 (50.00)
Participant currently has fishy-smelling abnormal discharge				
No (%)	34 (87.18)	12 (92.31)	11 (91.67)	11 (78.57)
Yes (%)	5 (12.82)	1 (7.69)	1 (8.33)	3 (21.43)
Have you been treated for STIs in last 12 months				
No (%)	34 (89.47)	11 (84.62)	10 (90.91)	13 (92.86)
Yes (%)	4 (10.53)	2 (15.38)	1 (9.09)	1 (7.14)
Ever treated for BV in the last 12 months				
No (%)	37 (94.87)	13 (100.0)	10 (83.33)	14 (100.0)
Yes (%)	2 (5.13)	0 (0.00)	2 (16.67)	0 (0.00)
Age of debut sex				
< 16 years (%)	5 (12.82)	3 (23.08)	0 (0.00)	2 (14.29)
≥ 16 years (%)	34 (87.18)	10 (76.92)	12 (100.0)	12 (85.71)
Current male partners				
< 2 (%)	33 (84.62)	12 (92.31)	11 (91.67)	10 (71.43)
≥ 2 (%)	6 (15.38)	1 (7.69)	1 (8.33)	4 (28.57)
Male partners in the last 12 months				
< 2 (%)	26 (66.67)	9 (69.23)	7 (58.33)	10 (71.43)
≥ 2 (%)	13 (33.33)	4 (30.77)	5 (41.67)	4 (28.57)
New male partners in the last 12 months				
< 2 (%)	35 (89.74)	12 (92.31)	11 (91.67)	12 (85.71)
≥ 2 (%)	4 (10.26)	1 (7.69)	1 (8.33)	2 (14.29)
Condom use in the last 12 months				
Always (%)	6 (15.38)	2 (15.38)	2 (16.67)	2 (14.29)
Never (%)	12 (30.77)	6 (46.15)	4 (33.33)	2 (14.29)
Sometimes (%)	21 (53.85)	5 (38.46)	6 (50.00)	10 (71.43)
Do you practice vaginal douching				
No (%)	39 (100.0)	13 (100.0)	12 (100.0)	14 (100.0)
Yes (%)	0 (0.00)	0 (0.00)	0 (0.00)	0 (0.00)
Use soap to wash vagina to make it smell good?				
No (%)	22 (56.41)	8 (61.54)	10 (83.33)	4 (28.57)
Yes (%)	17 (43.59)	5 (38.46)	2 (16.67)	10 (71.43)
Buds and/or hyphae (yeast)				
Negative (%)	36 (92.31)	12 (92.31)	11 (91.67)	13 (92.86)
Positive (%)	3 (7.69)	1 (7.69)	1 (8.33)	1 (7.14)
vaginal sex in a week for the last 12 months				
0–3 times (%)	30 (83.33)	12 (92.31)	10 (100.0)	8 (61.54)
> 3 times (%)	6 (16.67)	1 (7.69)	0 (0.00)	5 (38.46)
Continuing to use injectable contraceptives				
No (%)	2 (5.41)	1 (8.33)	1 (8.33)	0 (0.00)
Yes (%)	35 (94.59)	11 (91.67)	11 (91.67)	13 (100.0)

^a^ Group 2 and 3 participants were significantly more likely (p = 0.0001) to have vaginal abnormal discharge compared to Group 1 participants

Fourteen women (36%) reported abnormal discharge (Table 2). Group 2 and 3 BV participants were significantly more likely to have vaginal abnormal discharge compared to Group 1 participants (50% vs. 7.69%, *p* = 0.0001, Table 2). Four (11%) women had been treated for STI in the past 12 months and only two (5%) (in Group 2) reported ever being treated for BV. Most participants (87%) had sexual debut aged ≥16 years. Six women (15%) reported ≥2 current male partners, 13 (33%) reported ≥2 male partners in the last 12 months and among them, 4 (10%) had ≥2 new male partners. Absence or inconsistent condom use in the last 12 months was reported by 85% of the women. Vaginal douching was not reported by anyone but use of soaps to wash the vagina was reported by 17 (44%) women. At the end of follow-up, HIV seroconversion occurred in one participant in Group 3.

### Clinical outcome

Six women did not show up for one to three visits during follow-up for a total of 11 missing samples out of 226 (Additional file 1: Figure S5). Discordance was observed between Nugent and Hay-Ison score for 8 out of 215 samples but this did not affect the diagnosis of cure or relapse. Of the 13 women in the healthy arm, 4 (31%) developed BV infection during follow-up of which 2 were diagnosed at day 30 (visit 2). There was no significant difference in cure and relapse rate between females receiving a combination of antibiotics and probiotic (Group 2) compared to women treated with antibiotics only (Group 3). The cure rate at visit 2 (1 month follow-up) was 42% (5/12) and 36% (5/14) in Groups 2 and 3, respectively while the relapse rate in women initially cured at visit 2 in these two groups was 40% (2/5) and 25% (1/4), respectively (1 cured participant in Group 3 withdrew on visit 5). The cure rate after 6 months was 25% (3/12) for both Group 2 and 3 (considering two women were lost to follow-up in Group 3) (Fig. 2 and Additional file 1: Figure S5).

### Factors associated with the relative risk (RR) of BV during follow-up

Relative to women in Group 1, the women in Group 2 (RR: 3.60, 95% CI: 1.32–9.81, *p* = 0.0123) and Group 3 (RR: 4.39, 95% CI: 1.59–12.2, *p* = 0.0044) and those reporting previous treatment for BV in the past 12 months (RR: 2.31, 95% CI: 1.16–4.60, *p* = 0.0173) had a higher relative risk for BV (Additional file 1: Table S3). No associations were observed with other factors such as tobacco and alcohol use, number of partners, number of partners in the past 12 months, condom use, frequency of vaginal sex, vaginal douching, etc. (Additional file 1: Table S3).

### Colonisation with lactobacilli

**Fig. 3:**
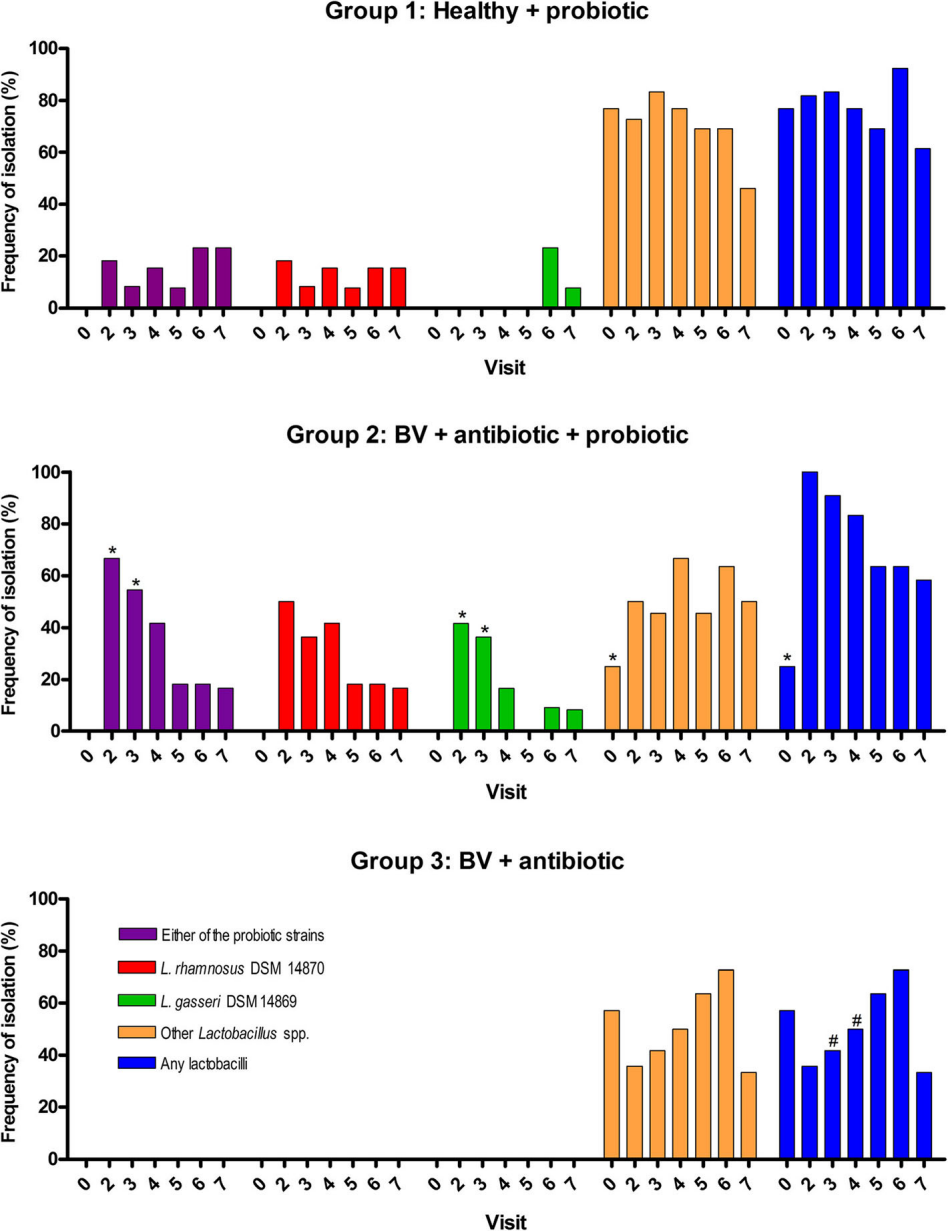
Frequency of isolation of *Lactobacillus* in different treatment groups. Presence of *L. gasseri* DSM 14869, *L. rhamnosus* DSM 14870 and other lactobacilli was evaluated in vaginal swabs before treatment (visit 0) and follow-up (visits 2 to 7). The frequency of isolation (y axis) was determined as the percentage of samples positive for probiotic strains or other lactobacilli on the total number of samples for each visit and each group. *Significantly different (*P* <  0.05, Fisher’s exact test) from Group 1 and 3, and # significantly different (P <  0.05, Fisher’s exact test) from Group 1 and 2 for corresponding visits

During the follow-up, the proportion of women colonized with *L. gasseri* DSM 14869 or *L. rhamnosus* DSM 14870 as well as the percentage of samples positive for both strains (frequency of isolation for visits 2 to 7) was higher in Group 2 than Group 1 (*p* = 0.041 and *p* = 0.007, respectively) (Table 3, Additional file 1: Figure S5 A and B). Immediately following administration of probiotic capsules daily for 30 days (visit 2), 67% (8/12) of women were colonised with *L. gasseri* DSM 14869 or *L. rhamnosus* DSM 14870 (or both) in Group 2 compared to 18% (2/11) in Group 1 (*p* = 0.011) (Fig. 3, Additional file 1: Figure S5 A and B). The two strains could still be observed in 42% (5/12) participants of Group 2 on visit 4 (Fig. 3, Additional file 1: Figure S5 B). *L. rhamnosus* DSM 14870 was slightly more frequently isolated than *L. gasseri* DSM 14869 in both Group 1 and Group 2 but the difference was not significant (*p* = 0.16) (Table 3, Additional file 1: Figure S5 A and B). No significant difference in the frequency of isolation of any lactobacilli was observed between Group 1 and 2 (*p* = 0.843) but both groups had a higher number of vaginal samples positive for lactobacilli than Group 3 (*p* <  0.001) (Table 3, Fig. 3).

**Table 3:** Colonisation with lactobacilli: number of women colonised and frequency of isolation during follow-up

Group	Colonisation and isolation frequency of probiotic strains and other lactobacilli				
	Group	Women colonised	*P*-value^a^	Frequency of isolation (%)^b^	*P*-value^a^
Either of the probiotic strains	Group 1	5/13 (39%)		12/75 (16%)	
	Group 2	10/12 (83%)	*p* = 0.041 (vs Gr. 1)*	25/69 (36%)	*p* = 0.007 (vs Gr. 1)*
	Group 3	0/14 (0%)	*p* = 0.0159 (vs Gr. 1)*	0/71 (0%)	*p* = 0.0003 (vs Gr. 1)*
			*p* = 0.0001 (vs Gr. 2)*		*p* < 0.0001 (vs Gr. 2)*
*L. rhamnosus*	Group 1	3/13 (23%)		10/75 (13%)	
	Group 2	9/12 (75%)	*p* = 0.017 (vs Gr. 1)*	21/69 (30%)	*p* = 0.015 (vs Gr. 1)*
	Group 3	0/14 (0%)	*P* > 0.05 (vs Gr. 1)	0/71 (0%)	*p* = 0.0015 (vs Gr. 1)*
			*P* = 0.0001 (vs Gr. 2)*		*p* < 0.0001 (vs Gr. 2)*
*L. gasseri*	Group 1	2/13 (15%)		4/75 (5%)	
	Group 2	8/12 (67%)	*p* = 0.015 (vs Gr. 1)*	13/69 (19%)	*p* = 0.018 (vs Gr. 1)*
	Group 3	0/14 (0%)	*P* > 0.05 (vs Gr. 1)	0/71 (0%)	*p* > 0.05 (vs Gr. 1)
			*p* = 0.0003 (vs Gr. 2)*		*p* < 0.0001 (vs Gr. 2)*
Other lactobacilli	Group 1	12/13 (92%)		53/75 (71%)	
	Group 2	12/12 (100%)	*p* > 0.05 (vs Gr. 1)	39/69 (57%)	*p* > 0.05 (vs Gr. 1)
	Group 3	12/14 (86%)	*p* > 0.05 (vs Gr. 1)	35/71 (49%)	*p* = 0.011 (vs Gr. 1)*
			*p* > 0.05 (vs Gr. 2)		*p* > 0.05 (vs Gr. 2)
Any lactobacilli	Group 1	13/13 (100%)		59/75 (78%)	
	Group 2	12/12 (100%)	*p* > 0.05 (vs Gr. 1)	53/69 (77%)	*p* > 0.05 (vs Gr. 1)
	Group 3	12/14 (86%)	*p* > 0.05 (vs Gr. 1)	35/71 (49%)	*p* = 0.0003 (vs Gr. 1)*
			*p* > 0.05 (vs Gr. 2)		*p* = 0.0009 (vs Gr. 2)*

^a^Significant difference by Fisher’s exact test (two tailed) indicated by an asterisk (*)

^b^The frequency of isolation was determined as the number of samples positive for probiotic strains or other lactobacilli on the total number of samples for each group during the entire follow-up (visits 2-7)

None of the *Lactobacillus* isolated at visit 0 (before administration of probiotics) from Group 1 and 2 or at visits 0 to 7 from Group 3 (not receiving the capsules) tested positive for *L. gasseri* DSM 14869 or *L. rhamnosus* DSM 14870 confirming the specificity of the PCR detection method (Fig. 3 and Additional file 1: Figure S5).

**Fig. 4:**
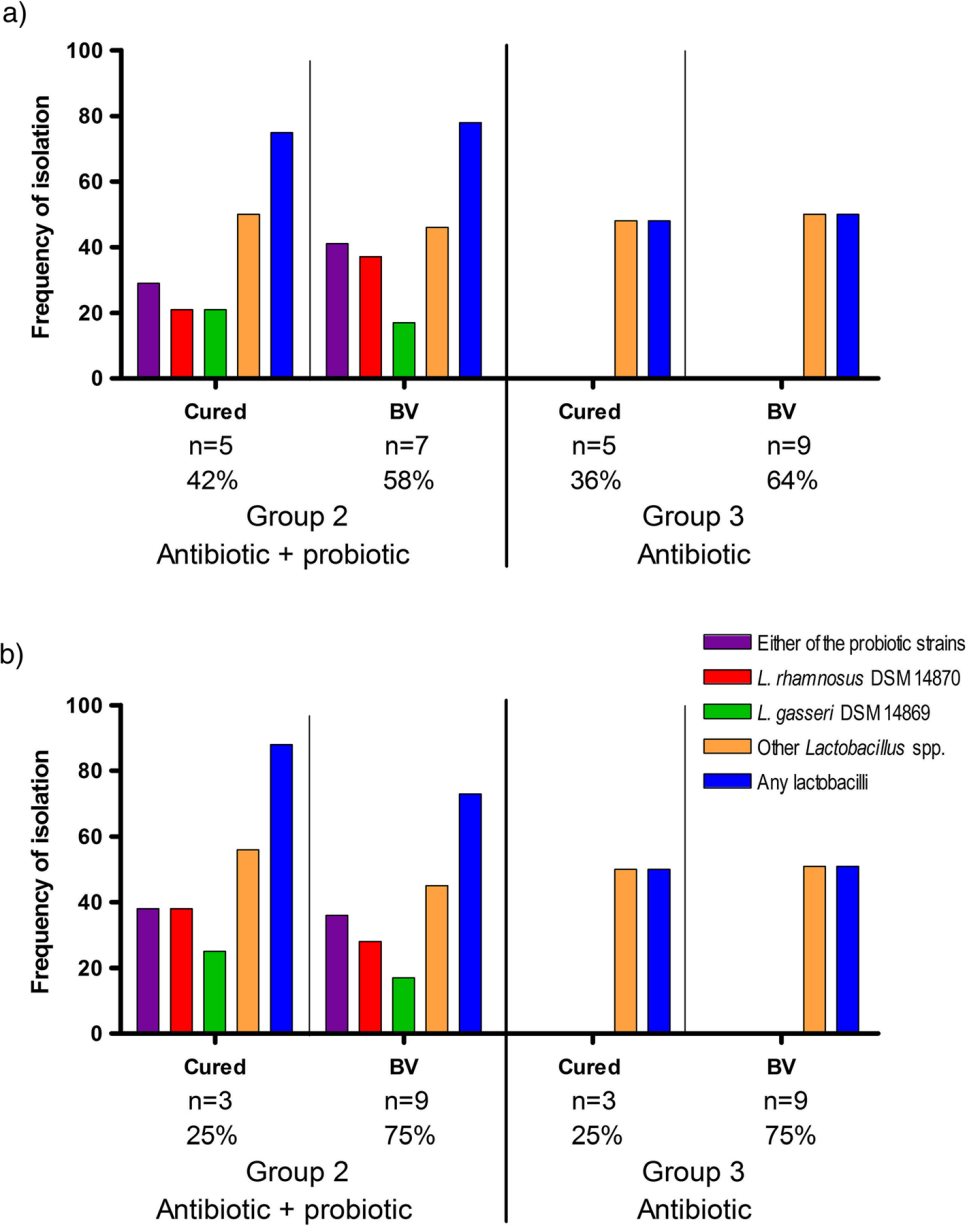
Association between the frequency of isolation of probiotic strains and other lactobacilli with the 1-month (**a**) and 6-month (**b**) cure rates. The frequency of isolation (y axis) was determined as the percentage of samples positive for probiotic strains and other lactobacilli on the total number of samples for each group (cured or BV positive) during the entire follow up. No statistical difference in frequency of isolation of probiotic strains or other lactobacilli was observed between cured and BV-positive women within Group 2 (antibiotic + probiotic) and Group 3 (antibiotic)

In Group 2, the frequency of isolation of *L. gasseri* DSM 14869 or *L. rhamnosus* DSM 14870 during the entire follow-up (visits 2 to 7) was similar (*p* >  0.05) between cured women and women with BV (based on the 1-month and 6-month cure rates) (Fig. 4a and b). Furthermore, the frequency of isolation of both probiotic strains was similar (*p* > 0.05) between the three women cured for the entire study (6 out of 16 samples, 38%) and the two women with relapse (2 out 12 samples, 17%) (data not shown). These results suggest that colonisation with administered strains is not significantly associated with cure and prevention of relapse. Furthermore no association was observed between colonisation by other lactobacilli (or any lactobacilli) and cure in both Group 2 and 3 (Fig. 4). Similar results were obtained when calculating the frequency of isolation for each woman individually and comparing the mean between cured and women with BV (Additional file 1: Figure S6).

## Discussion

In the present study, we reported a low BV cure rate over the course of 6 months in South African women treated with standard antibiotic treatment. Furthermore, supplementation with vaginal probiotic capsules resulted in colonisation of the vagina by the *Lactobacillus* strains (*L. rhamnosus* DSM 14870 and *L. gasseri* DSM 14869) contained in the capsules but did not have any significant effect on BV cure rate or prevention of relapses.

In resource-limited settings, WHO recommends a syndromic diagnostic approach for the management of sexually transmitted infection and symptomatic BV. Diagnosis is often missed because symptoms are frequently absent or non-specific, and the microscopic methods necessary for diagnosis by Amsel criteria or Nugent scoring are typically not available [16]. In the present study, the prevalence of BV diagnosed by Nugent scoring (38%) was similar with our previous observations in the Soweto population (35%) [26]. Of the women enrolled, only 50% reported abnormal vaginal discharge which is in line with a study in Uganda reporting that more than 50% of the BV cases diagnosed by Nugent score were asymptomatic [12]. Only women using injectable contraceptive (depot medroxyprogesterone acetate or norethisterone enanthate) were enrolled in order to reduce heterogeneity in our small sample size population. Some studies reported that the use of injectable progestin is associated with a decreased risk of BV (and paradoxically increased HIV acquisition) but the reported BV frequency in the present population was still high [42].

In South Africa, BV is treated according to national guideline for managing vaginal discharge syndrome with 2 g of metronidazole in a single oral dose (in combination with cefixime and doxycycline for treatment of STI). A single dose might be less efficacious and lead to more recurrence than the generally recommended 7-day course metronidazole (400–500 mg) [43]. We observed a very low cure rate in South African women receiving a single oral dose of metronidazole (1-Month and 6-Month cure rates of 36 and 25%) compared to cure rates reported in Caucasian females receiving a 7-day metronidazole regime (1-Month and 6-Month cure rates of 90 and 42%, respectively) [9]. Other studies have also reported a low cure rate and recurrence of BV in sub-Saharan Africa receiving either a single dose or a 7-day course of metronidazole (cure rates of 40 and 28% at 1- to 3- months follow-up respectively for both treatments) [12, 29, 30]. In a recent study conducted in Cape Town, 69% of women treated with one single dose of metronidazole still had BV two months after treatment (first follow-up visit) indicating high recurrence rates or a poor response to the single-dose metronidazole regimen [44].

In a previous double-blind, randomised, placebo-controlled clinical study in Scandinavia, we showed that adjunct treatment with capsules containing *L. rhamnosus* DSM 14870 and *L. gasseri* DSM 14869 after antibiotic therapy (seven-day course of daily 2% vaginal clindamycin cream) could increase the cure rate at the end of the 6-months follow-up by nearly 20% (from 46 to 65%) compared to placebo treated women [27]. We have also demonstrated that a more aggressive antibiotic treatment (2% vaginal clindamycin 7 days together with 300 mg of oral clindamycin followed around 2–3 weeks later by vaginal 0.75% metronidazole gel) along with *Lactobacillus* administration could provide a long lasting cure against BV (12-month cure rate > 65%) [26, 28]. In the present study, the initial cure rate (1-month) was slightly higher in women receiving antibiotic and probiotic compared to women receiving antibiotic only (42 vs 36%) but we did not observe that supplement with probiotic capsules significantly increased the cure rate or prevent the number of relapse. These results could be due to the milder antibiotic treatment and low initial cure rate compared to our studies in Scandinavia. In Norwegian women, self-administration of vaginal capsules did not improve the efficacy of BV therapy during the initial first month (> 75% cure rate in both antibiotic and probiotic-supplemented groups) but significantly improved the time to relapse [27]. Previous studies have also evaluated supplementation of metronidazole therapy with oral probiotic in Africa. In a study performed in 2006 in Nigeria, oral *L. rhamnosus* GR-1 and *L. reuteri* RC-14 twice daily for 30 days resulted in a 88% cure in the antibiotic/probiotic group compared to 40% in the antibiotics/placebo at 30-days follow-up [29]. However these results could not be repeated in a following study performed in Tanzania, where supplementation of metronidazole treatment (10 days, 400 mg twice daily) with oral probiotic *L. rhamnosus* GR-1 and *L. reuteri* RC-14 (twice daily for 6 months) did not enhance the cure of BV among HIV infected women [30]. In our study, the lack of statistical significance between arms may also be because this was a pilot study with low numbers of enrolment which may have affected the power to detect differences. The rate of incident BV in the healthy group, and the failure in the two antibiotic groups provides appropriate data from which to generate an estimate of how large a true clinical trial would need to be.

Interestingly, 31% of the women from the healthy group (Group 1) developed BV during the 6-month follow-up period which is near the calculated prevalence in the population (35%). Women previously treated for BV were more likely to test positive for BV but contrary to published studies [12, 45], we could not find a statistically significant association between BV prevalence and a number of socio-economic and sexual behavioral risk factors including numbers of sex partners, lack of condom use and frequency of sexual intercourse both when analyzing all three groups together or only treated groups (Group 2 and 3). Recall bias may have affected some of the risk behavior questions such as condom use and number of sex acts in the past 12 months limiting associations between groups. Furthermore although data on new sexual partner were recorded at enrolment, this information was not recorded during the follow-up period. From our other study in Scandinavia, this information was the strongest prediction for relapse but all regular partners were given antibiotic treatment [26]. The generalisability of our findings is also limited by the low sample size, insufficient power and our strict eligibility criteria of requiring women to be on one of only two contraceptives and to read and understand English.

As previously reported, we observed that women with BV and treated with antibiotics are colonised better by *L. rhamnosus* DSM 14870 and *L. gasseri* DSM 14869 than women with no BV [28]. The imbalance of the vaginal microbiota, antibiotic treatment or absence of competitive resident lactobacilli might promote the colonisation by exogenous lactobacilli. We have previously shown that either *L. rhamnosus* DSM 14870 or *L. gasseri* DSM 14869 could persist in the vagina of 80% of Scandinavian women treated with antibiotics and vaginal capsules at least two weeks after stopping the administration [28]. In comparison, we observed that either *L. rhamnosus* DSM 14870 or *L. gasseri* DSM 14869 colonise a similar amount of South African women (83%) with BV although the frequency of isolation during follow-up was slightly lower than in Scandinavia (49% vs 36% of samples). Contrary to previous results obtained in Scandinavia, no correlation was observed between colonisation with the two *Lactobacillus* strains and with cure and prevention of relapse [28]. This could be due to the less aggressive antibiotic treatment or lower adherence to self-administration of capsules in South Africa compared to Scandinavia.

The culture-based and PCR identification method used in this study may favor the identification of the *Lactobacillus* species dominating the microbiota which might explain why the administered *Lactobacillus* strains were only sporadically detected for some women. Therefore, our results need to be confirmed using molecular methods for quantification of *L. rhamnosus* DSM 14870 and *L. gasseri* DSM 14869. Furthermore, the impact of administration of capsules on the whole vaginal microbiome should also be measured to better understand how the administered *Lactobacillus* strains may help in restoring the normal microbiota and reduce relapse of BV.

## Conclusion

We observed low initial cure rates of BV from a stat dose of metronidazole, suggesting that the syndromic approach to management of vaginal discharge, which is the current policy in South Africa, is sub-optimal for BV treatment. The low initial BV cure rates may have undermined the ability of *L. rhamnosus* DSM 14870 or *L. gasseri* DSM 14869 to prevent recurrence. The use of probiotic capsules as an adjunct therapy to improve antibiotic treatment requires further investigation possibly by using an alternative antibiotic regime in a larger cohort. For populations with high BV prevalence and high HIV and STI incidence, more effective treatment strategies for BV are urgently needed. The development of innovative treatment strategies that effectively treat initial episodes of BV and prevent rapid recurrence deserves further scrutiny. Furthermore, altering the vaginal microbiome with antibiotics and probiotics is one of the new directions being pursued for HIV prevention [46]. The efficacy of tenofovir disoproxyl fumarate vaginal gel was shown to be higher in *Lactobacillus*-dominant women than women with no lactobacilli, suggesting that an intervention to maintain a *Lactobacillus*-dominant microbiome might optimize the efficacy of tenofovir vaginal gel to prevent HIV infection [47]. Colonisation by *L. rhamnosus* DSM 14870 and *L. gasseri* DSM 14689 strains suggests that these strains could be used for modification of the microbiome and for delivery of a microbicides targeting HIV.

## Additional file

Additional file 1: **Table S1.** Primers and amplified DNA fragments. **Table S2.** *Lactobacillus* strains used to test the primers specificity. **Table S3.** Factors associated with bacterial vaginosis during follow-up. **Figure S1.** Clustered regularly interspaced short palindromic repeats (CRISPRs, in yellow) alternate with spacers of different sequence but similar length. **Figure S2.** Amplification of the *Lactobacillus* 16S rRNA gene segment with the genus-specific primers L and R. **Figure S3.** Specificity of the multiplex PCR for the detection of *L. rhamnosus* DSM 14870. **Figure S4.** Specificity of the multiplex PCR for the detection of *L. gasseri* DSM 14869. **Figure S5** Isolation of *L. rhamnosus* DSM 14870 and *L. gasseri* DSM 14869 in vaginal samples. **Figure S6** Association between the mean frequency of isolation of probiotic strains and other lactobacilli with the A) 1-month and B) 6-month cure rates. (PDF 3297 kb)

## Data Availability

The datasets used and/or analyzed during the current study are available from the corresponding author on reasonable request.

## Abbreviations

BV: Bacterial vaginosis
HIV: Human Immunodeficiency Virus
IQR: interquartile range
STI: Sexually transmitted infections
