# Supplementary material for: An exploratory pilot study evaluating the supplementation of standard antibiotic therapy with probiotic lactobacilli in south African women with bacterial vaginosis
Source: BMC Infect Dis. 2019 Sep 18;19:824. doi: 10.1186/s12879-019-4425-1 (PMC6751625; doi:10.1186/s12879-019-4425-1)
Supplement: Supplementary file 1 — Table S1. Primers and amplified DNA fragments. Table S2. Lactobacillus strains used to test the primers specificity. Table S3. Factors associated with bacterial vaginosis during follow-up. Figure S1. Clustered regularly interspaced short palindromic repeats (CRISPRs, in yellow) alternate with spacers of different sequence but similar length. Figure S2. Amplification of the Lactobacillus 16S rRNA gene segment with the genus-specific primers L and R. Figure S3. Specificity of the multiplex PCR for the detection of L. rhamnosus DSM 14870. Figure S4. Specificity of the multiplex PCR for the detection of L. gasseri DSM 14869. Figure S5 Isolation of L. rhamnosus DSM 14870 and L. gasseri DSM 14869 in vaginal samples. Figure S6 Association between the mean frequency of isolation of probiotic strains and other lactobacilli with the A) 1-month and B) 6-month cure rates. (PDF 3297 kb) [file 12879_2019_4425_MOESM1_ESM.pdf]

Table S1 Primers and amplified DNA fragments

Primers	Sequence	Target sequence	Size of PCR product (bp)	References
L	5´-TGGAAACAGGTGCTAATACCG-3´	*Lactobacillus*16 SRNA	230	McOrist *et al*. 2002
R	5´-CCATTGTGGAAGATTCCC-3´			
P0	5’-GAGAGTTTGATCCTGGCTCAG-3’	Eubacterial 16 SRNA	1 507	Di Cello and Fani, 1996
P6	5’-CTACGGCTACCTTGTTACGA-3’			
scr-Rhmn-2Fw	5’- AAAGTCGTGTATATGTAGCCGG -3’	CRISPR array	562	This study
scr-Rhmn-2Rv	5’- GAACCGTCTCGTCTTCCAATAG -3’			
scr-gass-1Fw	5’-CCTATTGTCGAACCCTTACGAA-3	CRISPR array	392	This study
scr-gass-1Rv	5’- GTGCTGAAATGCTTGGTGTAG -3’			

Table S2 *Lactobacillus* strains used to test the primers specificity

**Strain**	**Origin of the strain**	**Reference**
L. gasseri S1011RA2	Vaginal, South Africa	Penharkar *et al*. 2013 [a]
L. gasseri S1013RA1	Vaginal, South Africa	Penharkar *et al*. 2013
L. gasseri S1040RAS3	Vaginal, South Africa	Penharkar *et al*. 2013
L. gasseri S1025RA1	Vaginal, South Africa	Penharkar *et al*. 2013
L. gasseri S1184RA1	Vaginal, South Africa	Penharkar *et al*. 2013
L. gasseri S1196RA1	Vaginal, South Africa	Penharkar *et al*. 2013
L. gasseri 16R5	Vaginal, Sweden	Larsson *et al*. 2011 [b]
L. gasseri 6M7	Vaginal, Sweden	Larsson *et al*. 2011
L. gasseri 15M9	Vaginal, Sweden	Larsson *et al*. 2011
L. gasseri 7B1	Vaginal, Sweden	Larsson *et al*. 2011
L. gasseri 20M39	Vaginal, Sweden	Larsson *et al*. 2011
L. gasseri 6M9	Vaginal, Sweden	Larsson *et al*. 2011
L. gasseri 20M37	Vaginal, Sweden	Larsson *et al*. 2011
L. gasseri 16B1	Vaginal, Sweden	Larsson *et al*. 2011
L. gasseri E9B4-1	Intestinal, Estonia	Koll *et al*. 2010 [c]
L. gasseri 177-3	Intestinal, Estonia	Koll *et al*. 2010
L. gasseri E16B7	Intestinal, Estonia	Koll *et al*. 2010
L. gasseri E101G2-4-2	Intestinal, Estonia	Koll *et al*. 2010
L. rhamnosus 16.5.2-1	Vaginal, Sweden	Penharkar *et al*. 2015 [d]
L. rhamnosus 17.3.2-3	Vaginal, Sweden	Penharkar *et al*. 2015
L. rhamnosus 23.2.2-1	Vaginal, Sweden	Penharkar *et al*. 2015
L. rhamnosus GG ATCC 53103	Intestinal, US	Gorbach and Goldin, 1983
L. rhamnosus 35-DLB-5	Oral, Estonia	Koll *et al*. 2008 [e]
L. rhamnosus 14-DLB-8	Oral, Estonia	Koll *et al*. 2008
L. rhamnosus 19-DLB-2A	Oral, Estonia	Koll *et al*. 2008
L. rhamnosus 19-DLB-5	Oral, Estonia	Koll *et al*. 2008
L. rhamnosus 21-DLB-5B	Oral, Estonia	Koll *et al*. 2008
L. rhamnosus 21-DLB-7	Oral, Estonia	Koll *et al*. 2008
L. rhamnosus 23-DLB-2	Oral, Estonia	Koll *et al*. 2008
L. rhamnosus 23-DLB-5	Oral, Estonia	Koll *et al*. 2008
L. rhamnosus 23-DLB-6	Oral, Estonia	Koll *et al*. 2008
L. mucosae S1002RA1	Vaginal, South Africa	Pendharkar *et al*. 2013
L. paracasei S1002RA4	Vaginal, South Africa	Pendharkar *et al*. 2013
L. coleohominis S1003RA4S	Vaginal, South Africa	Pendharkar *et al*. 2013
L. crispatus S1003RA1B	Vaginal, South Africa	Pendharkar *et al*. 2013
L. crispatus S1040RAS1	Vaginal, South Africa	Pendharkar *et al*. 2013
L. crispatus S1098RAR1	Vaginal, South Africa	Pendharkar *et al*. 2013
L. crispatus S1056RA1	Vaginal, South Africa	Pendharkar *et al*. 2013
L. crispatus S1083RA1	Vaginal, South Africa	Pendharkar *et al*. 2013
L. crispatus S1178RA1	Vaginal, South Africa	Pendharkar *et al*. 2013
L. crispatus S1181RA1	Vaginal, South Africa	Pendharkar *et al*. 2013
L. crispatus S1182RA1	Vaginal, South Africa	Pendharkar *et al*. 2013
L. crispatus S1185RA3	Vaginal, South Africa	Pendharkar *et al*. 2013
L. crispatus S1197RA3	Vaginal, South Africa	Pendharkar *et al*. 2013
L. vaginalis S1004RA1	Vaginal, South Africa	Pendharkar *et al*. 2013
L. vaginalis S1008RA1	Vaginal, South Africa	Pendharkar *et al*. 2013
L. vaginalis S1040RAS2	Vaginal, South Africa	Pendharkar *et al*. 2013
L. vaginalis S1098RAP1	Vaginal, South Africa	Pendharkar *et al*. 2013

L. vaginalis S1131RAB1	Vaginal, South Africa	Pendharkar *et al*. 2013
L. iners S1183BA1	Vaginal, South Africa	Pendharkar *et al*. 2013
L. iners S1006BA2	Vaginal, South Africa	Pendharkar *et al*. 2013
L. iners S1008BA4	Vaginal, South Africa	Pendharkar *et al*. 2013
L. iners S1116BA1	Vaginal, South Africa	Pendharkar *et al*. 2013
L. ruminis S1002RA9	Vaginal, South Africa	Pendharkar *et al*. 2013
L. ruminis S1174RA1	Vaginal, South Africa	Pendharkar *et al*. 2013
L. ruminis S1175RA1	Vaginal, South Africa	Pendharkar *et al*. 2013
L. jensenii S1014RA1	Vaginal, South Africa	Pendharkar *et al*. 2013
L. jensenii S1132RA1	Vaginal, South Africa	Pendharkar *et al*. 2013
L. jensenii S1185RA1	Vaginal, South Africa	Pendharkar *et al*. 2013
L. jensenii S1196RA3	Vaginal, South Africa	Pendharkar *et al*. 2013
L. plantarum TU196	Intestinal, Estonia and Sweden	Koll *et al*. 2010
L. fermentum 338-1-1	Intestinal, Estonia and Sweden	Koll *et al*. 2010
L. acidophilus 821-3	Intestinal, Estonia and Sweden	Koll *et al*. 2010

[a] Pendharkar S, Magopane T, Larsson PG, de Bruyn G, Gray GE, Hammarström L, Marcotte H. Identification and characterisation of vaginal lactobacilli from South African women. BMC Infect Dis. 2013 Jan 26; 13:43.

[b] Larsson PG, Brandsborg E, Forsum U, Pendharkar S, Andersen KK, Nasic S, Hammarström L, Marcotte H. Extended antimicrobial treatment of bacterial vaginosis combined with human lactobacilli to find the best treatment and minimize the risk of relapses. BMC Infect Dis. 2011 Aug 19; 11:223.

[c] Kõll P, Mändar R, Smidt I, Hütt P, Truusalu K, Mikelsaar RH, Shchepetova J, Krogh-Andersen K, Marcotte H, Hammarström L, Mikelsaar M. Screening and evaluation of human intestinal lactobacilli for the development of novel gastrointestinal probiotics. Curr Microbiol. 2010 Dec; 61(6):560-6.

[d] Pendharkar S, Brandsborg E, Hammarström L, Marcotte H, Larsson PG. Vaginal colonisation by probiotic lactobacilli and clinical outcome in women conventionally treated for bacterial vaginosis and yeast infection. BMC Infect Dis. 2015 Jul 3; 15:255.

[e] Kõll P, Mändar R, Marcotte H, Leibur E, Mikelsaar M, Hammarström L. Characterization of oral lactobacilli as potential probiotics for oral health. Oral Microbiol Immunol. 2008 Apr; 23(2):139-47.

Table S3 Factors associated with bacterial vaginosis during follow-up

Variable	n or median (IQR)	Univariate RR (95% CI)	Univariate p-value	Multivariate RR (95% CI)	Multivariate p-value
**Study Arm**					
Healthy + probiotic (Group 1)	13 (33.3)	Ref		Ref	
BV + antibiotic + probiotic (Group 2)	12 (30.8)	5.03 (1.90-13.4)	0.0012	3.60 (1.32-9.81)	0.0123
BV + antibiotic (Group 3)	14 (35.9)	4.90 (1.75-13.7)	0.0025	4.39 (1.59-12.2)	0.0044
**Age-Group**					
18-29 years	19 (48.7)	Ref			
30-40 years	20 (51.3)	0.92 (0.43-1.95)	0.8277		
**Education**					
Secondary	33 (84.6)	Ref			
Tertiary	6 (15.4)	0.87 (0.27-2.77)	0.8160		
**Employment**					
Yes	9 (23.1)	1.61 (0.77-3.35)	0.2084		
No	30 (76.9)	Ref			
**Housing type**					
Shack	5 (12.8)	0.99 (0.43-2.25)	0.9789		
Brick house	34 (87.2)	Ref			
**Alcohol use**					
Yes	24 (61.5)	0.86 (0.41-1.80)	0.6811		
No	15 (38.5)	Ref			
**Tobacco use**					
Yes	12 (30.8)	1.73 (0.84-3.55)	0.1377	1.88 (0.91-3.87)	0.0876
No	27 (69.2)	Ref		Ref	
**Abnormal fishy discharge**					
Yes	14 (35.9)	1.26 (0.62-2.56)	0.5241		
No	25 (64.1)	Ref			
**Ever treated for an STI in the past 12 months**					
Yes	4 (10.5)	0.25 (0.07-0.86)	0.0288	0.40 (0.17-0.96)	0.0413
No	34 (89.5)	Ref		Ref	
**Ever treated for BV in the past 12 months**					
Yes	2 (5.1)	2.07 (1.37-3.12)	0.0005	2.31 (1.16-4.60)	0.0173
No	37 (94.9)	Ref		Ref	

Variable	n or median (IQR)	Univariate		Multivariate	
		RR (95% CI)	p-value	RR (95% CI)	p-value
**Sex debut age**					
< 18 years	15 (38.5)	Ref			
=> 18 years	24 (61.5)	1.05 (0.47-2.35)	0.9085		
**Condom use**					
Consistent	6 (15.4)	Ref			
Inconsistent	33 (84.6)	0.57 (0.16-2.07)	0.3942		
**Frequency of weekly vaginal sex**	2 (1-3)	1.03 (0.77-1.39)	0.8270		
**Do you use soap to wash your vagina?**					
Yes	17 (43.6)	0.84 (0.38-1.89)	0.6808		
No	22 (56.4)	Ref			
**Number of partners**	1 (1-1)	0.77 (0.48-1.21)	0.2532		
**Number of partners in the past 12 months**	1 (1-2)	0.89 (0.56-1.40)	0.6088		

A) *L. rhamnosus* DSM 14870

cactgaaggtttttgatctgacatctacctgagactgccgcgtcgatctttgtcaatacccgagtgttcttgaactgattgat
ctgacatctacctgagaccaagcggtgggcaaactcacttcgtcatcagttcttgaactgattgatctgacatctacctgaga
ccttttttttcggcttgattgatatgttgcttgttcttgaactgattgatctgacatctacctgagaccaacggccagcgacag
cgcaacgcgtcaacgttcttgaactgattgatctgacatctacctgagactggcgcaacggaacaaggggcaaaatattggtt
cttgaactgattgatctgacatctacctgagacgtcttgccaaaccttgccgccatccttgtagttcttgaactgattgatct
gacatctacctgagaccgaaacaagtccggataggtatcattagcagttcttgaactgattgatctgacatctacctgagacg
gaaagtggtacaccgttgaggccgaacttgttcttgaactgattgatctgacatctacctgagactgcgcgtcgttttgattt
tgaagatgccgcgttcttgaactgattgatctgacatctacctgagacaacggtaagccacacaagcgactaacacgcgttct
tgaactgattgatctgacatctacctgagacctcggcgccgttgatggtcaatgtcagcgagttcttgaactgattgatctga
catctacctgagacaacgtgcaaaggtttcgccaactgcgtcatgttcttgaactgattgatctgacatctacctgagacggc
agcaattgccggctgttgttgatgttggttcttgaactgattgatctgacatctacctgagacgtaaatcgtgccggtattta
cttggctattgttcttgaactgattgatctgacatctacctgagacattaccacgcgttccgatggtaaaacattcgttcttg
aactgattgatctgacatctacctgagacacatacatttcttttacgtcatcgtgttcagttcttgaactgattgatctgaca
tctacctgagacaacggcactcgtgatgatggtatgcactacgttcttgaactgattgatctgacatctacctgagacggcga
cactggagcgctttcggttgctattgttcttgaactgattgatctgacatctacctgagacgctgtcagcgaagaccattgcg
ttcgcccggttcttgaactgattgatctgacatctacctgagaccgtggcatcggtcacggctttaaaattgccgttcttgaa
ctgattgatctgacatctacctgagactatttgggaaaatacaagcccatcataaaagttcttgaactgattgatctgacatc
tacctgagactgcaacgcgcgcacgtggatttgcacgactgttcttgaactgattgatctgacatctacctgagacagaccaa
ccggagagcgttaagcgtcattagttcttgaactgattgatctgacatctacctgagacagcttcaattgactattgcgggct
tccagagttcttgaactgattgatctgacatctacctgagaccg**aaagtcgtgtatatgtagccgg**caccctgagttcttgaa
ctgattgatctgacatctacctgagactgaaatgagtggtcaacttaaagaactttcgttcttgaactgattgatctgacatc
tacctgagaccaggaatgtacatccgcgcctcaatcaactgttcttgaactgattgatctgacatctacctgagacccagttt
ttggttgcttgtacatgctgtccgttcttgaactgattgatctgacatctacctgagacactcgttgcggtcatttctttgt
gcttgagttcttgaactgattgatctgacatctacctgagacaaagctctgaaagtggcaggactgtttctcgttcttgaact
gattgatctgacatctacctgagactcatctataagcaaatcgatctcaacattcgttcttgaactgattgatctgacatcta
cctgagacgaagggagaagggaatagttggaccttacagttcttgaactgattgatctgacatctacctgagaccaaggactg
tca**ctattggaagacgagacggttc**ttgaactgattgatctgacatctacctgagacttgcctacccaccagtcaaaacgtga
ggtagttcttgaactgattgatctgacatctacctgagaccggtgttttcacacagcctcttaatcaatcagttctcggc

B) *L. gasseri* DSM 14869

attggcaaattttagtaaataaaatgctgatttgacgggatttttttagtgtattctccacgtatgtggagatgatcctaccg
tattcccaattagcataggttgaaagtcgtattctccacgtatgtggagatgat**cctattgtcgaacccttacgaa**tacctag
gttagcgtattctccacgtatgtggagatgatcctaaagtttgctccccaaagtctggaatgtattcgtattctccacgtatg
tggagatgatcctaactggaagaagattgaagaaactatcaatcagtattctccacgtatgtggagatgatccttaattcaga
cattacggaggaatagaaaatgggtattctccacgtatgtggagatgatcctagcgatggaggtgctttcaatgagtgcattt
agtattctccacgtatgtggagatgatcccgttttgtttaattaacttacataattataatagtattctccacgtatgtggag
atgatccttg**ctacaccaagcatttcagcac**catcttgtagtattctccacgtatgtggagatgatccttgctttagaaatcc
attgaatcgcttctttatgtattctccacgtatgtggagatgatccttctcctttctaccctgtatcgtttcctccttgtat
tctccacgtatgtggagatgatcccactgctaaggctcgaaatttagctgatgtgaagtattctccacgtatgtggagatgat
ccttcctctctcaattttttcattctcttcttgtaggtattctccacgtatgtggagatgatccttattgagaggtgaaaacat
ggaaaaaagtaaagtattctccacgtatgtggagatgatcctgaagctaccgccgtcttggctgccaccaaagaggtattctc
cacgtatgtggagatgatcctggaataataagaatcaaatcgaatcaaatagagtattctccacgtatgtggagatgatccta
gaatcaaatagaatcataagaattattaatagtattctccacgtatgtggagatgatcctaaaaatcatggatttatcagaat
tagaaaagggtattctccacgtatgtggagatgatcctggcatcaggggcacaaatccaaacctataagagtattctccacgt
atgtggagatgatcccaagactcaatctgaagaagtaaaagatgaagtgtattctccacgtatgtggagatgatcctaagcca
gttcgaagggctactaactgggataagtattctccacgtatgtggagatgatcctaatgcccattccttgggataaaccatt
tccggtattctccacgtatgtggagatgatccttgcctgaatttgatcgttttaaggttggagatgtattctccacgtatgtg
gagatgatcccataaattcatggataaatttaccatgattcttgtattctccacgtatgtggagatgatcctgaaaaagaagc
ctgacgatgaaaaacaatttggtattctccacgtatgtggagatgatcctaaccaacttttgcgtcgaaatatccaaacaag
tattctccacgtatgtggaggactcctgatagacaaatatatcagcttttaggctaaagtaagaattttgtaggtataaaaaa
atgaaagttagatatgctggtgaaagtttttatagtgggttaggtttggcaaatggcaaggtatatgtagttttagataa

Figure S1 Clustered regularly interspaced short palindromic repeats (CRISPRs, in yellow) alternate with spacers of different sequence but similar length. A) *L. rhamnosus* DSM 14870 CRISPR locus, repeat region position between 992959 and 995240, B) *L. gasseri* DSM 14869 CRISPR locus 2 repeat region, position between 434451 and 436005. The primers sequences are indicated in red.

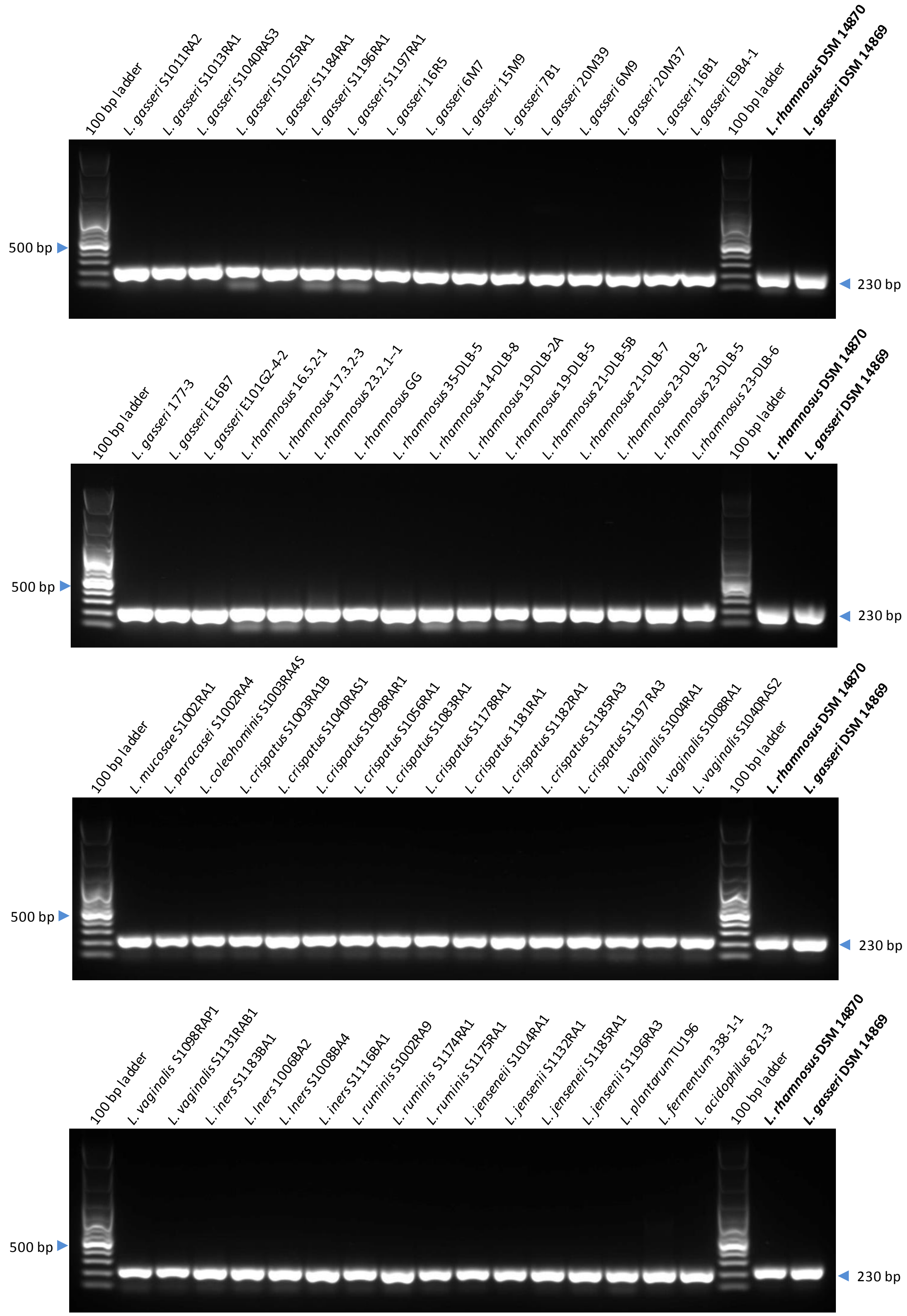

Figure S2 Amplification of the *Lactobacillus* 16S rRNA gene segment with the genus-specific primers L and R. The theoretical size of the PCR product is indicated on the right of the gel pictures. The origin of the strains used is described in Table S2.

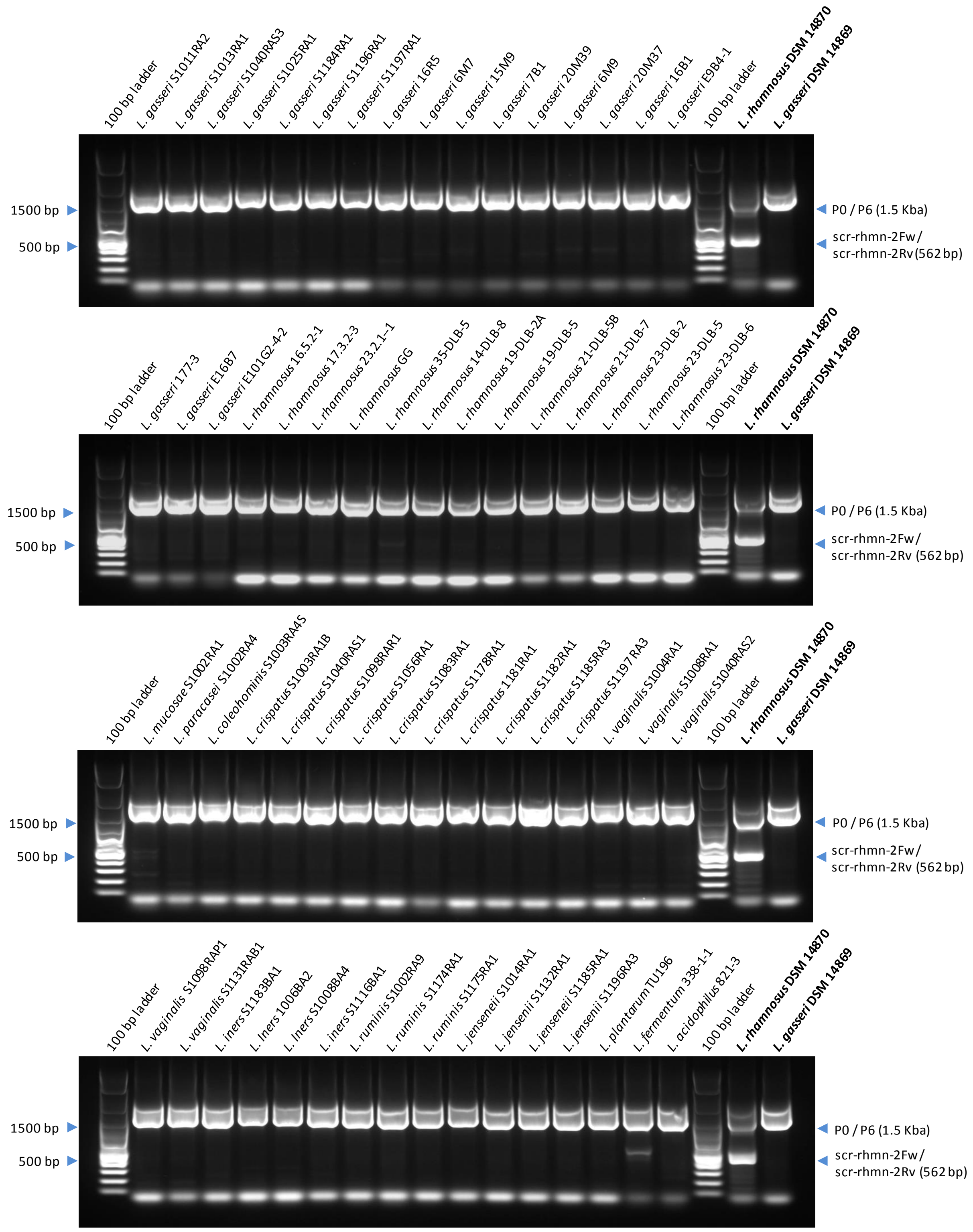

Figure S3 Specificity of the multiplex PCR for the detection of *L. rhamnosus* DSM 14870. The amplification of the 16S rRNA gene (internal control) with primers P0 and P6 resulted in a 1.5 Kb PCR product for all the *Lactobacillus* strains tested. The amplification of the CRISPR DNA segment with primers scr-rhmn-2Fw and scr-rhmn-2Rv resulted in a ~550 bp PCR product for *L. rhamnosus* DSM 14870 only. An additional light band was observed for *L. fermentum* 338-1-1 but at ~900 bp (lower panel, line 16). The theoretical size of the PCR products is indicated on the right of the gel pictures.

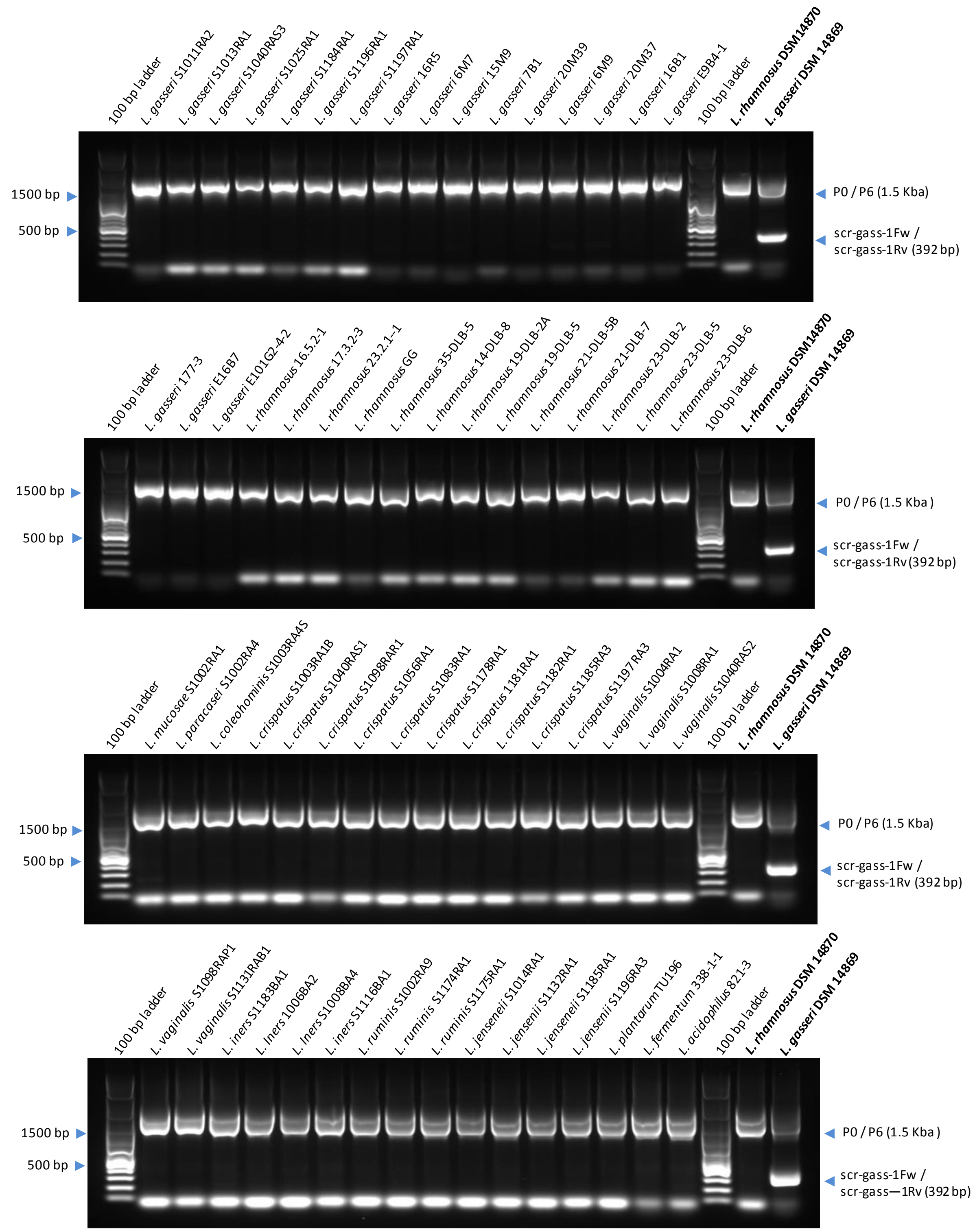

Figure S4 Specificity of the multiplex PCR for the detection of *L. gasseri* DSM 14869. The amplification of the 16S rRNA gene (internal control) with primers P0 and P6 resulted in a 1.5 Kb PCR product for all the *Lactobacillus* strains tested. The amplification of the CRISPR DNA segment with primers scr-gass-1Fw and scr-gass-1Rv resulted in a ~400 bp PCR product for *L. gasseri* DSM 14869 only. The theoretical size of the PCR products is indicated on the right of the gel pictures.

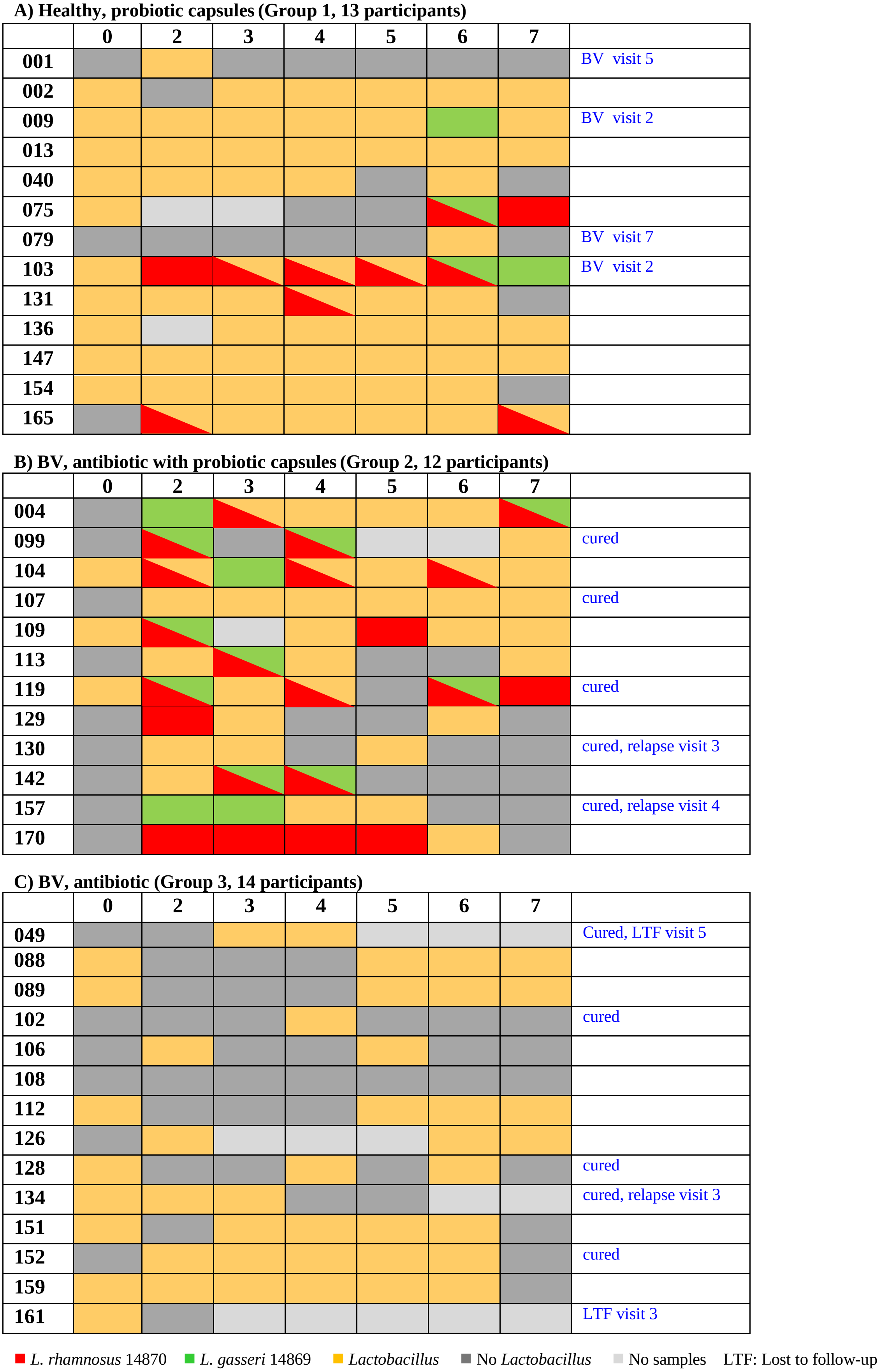

Figure S5 Isolation of *L. rhamnosus* DSM 14870 and *L. gasseri* DSM 14869 in vaginal samples.

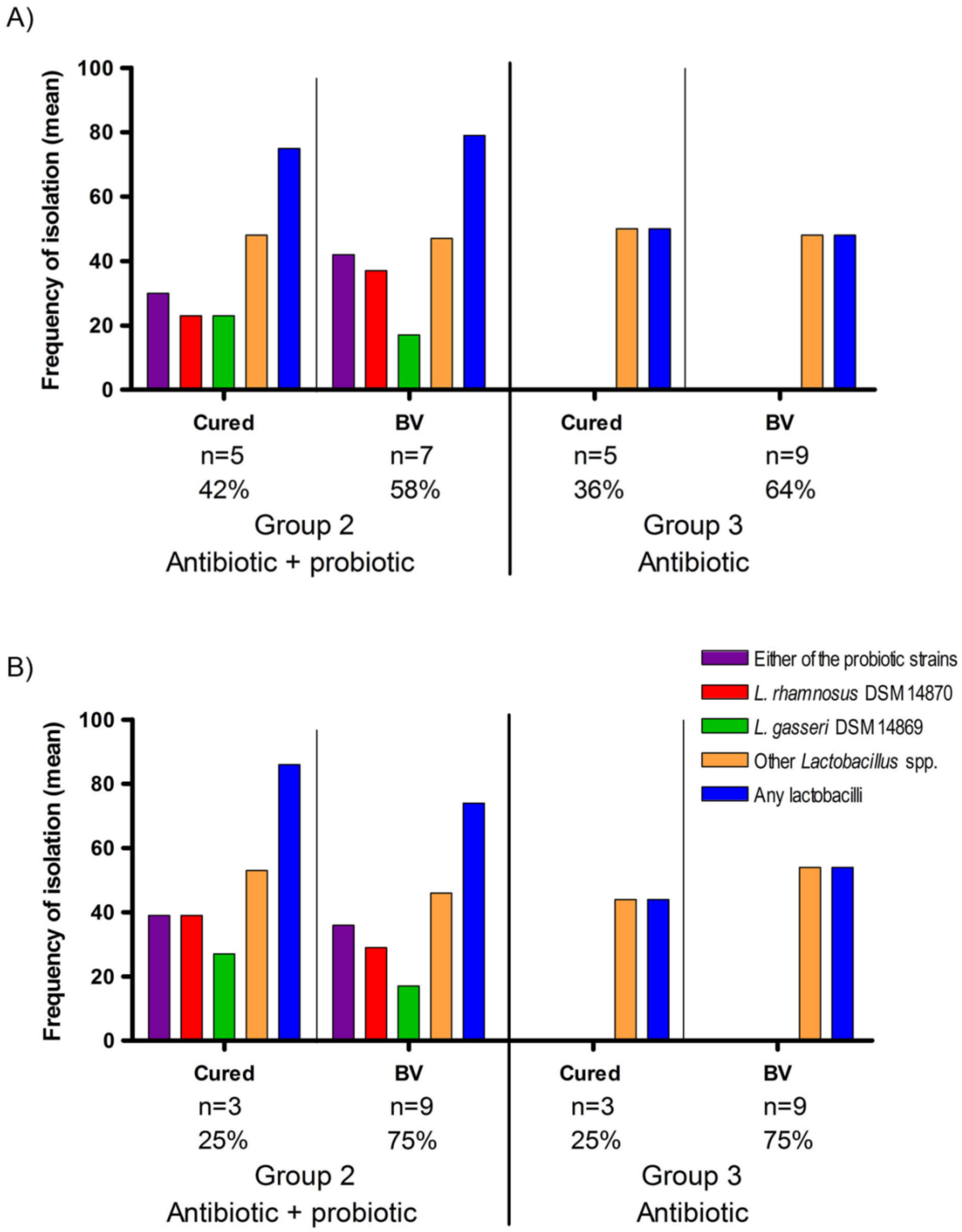

Figure S6 Association between the mean frequency of isolation of probiotic strains and other lactobacilli with the 1-month (A) and 6-month (B) cure rates. The frequency of isolation (y axis) was determined as the percentage of samples positive for probiotic strains and other lactobacilli on the total number of samples for each individual (cured or BV positive) during the entire follow up. The results are presented as a mean. No statistical difference in frequency of isolation of probiotic strains or other lactobacilli was observed between cured and BV positive women within Group 2 (antibiotic + probiotic) and Group 3 (antibiotic) using a t-test and a p-value cutoff of 0.05 (two tailed).
